# Unveiling the Feeding Behavior of *Tibraca limbativentris* (Hemiptera: Pentatomidae) on Rice Using an Electropenetrography Waveform Library

**DOI:** 10.1093/jisesa/ieaa064

**Published:** 2020-08-08

**Authors:** André Cirilo de S Almeida, Flávio Gonçalves de Jesus, José Alexandre F Barrigossi

**Affiliations:** 1 Federal Goiano Institute, Campus Urutaí, Rodovia Prof. Geraldo Silva Nascimento, Urutaí, GO, Brazil; 2 Embrapa Rice and Beans, Rodovia, Santo Antônio de Goiás, GO, Brazil

**Keywords:** electrical penetration graph, feeding site, rice stalk stink bug, *Oryza sativa*

## Abstract

The rice stalk stink bug, *Tibraca limbativentris* Stål, damages plant stalks while feeding, making it one of the most important rice pests in South America. Because the feeding behavior of *T. limbativentris* has not yet been studied in rice, we investigated *T. limbativentris* stylet penetration (probing) in rice stalks. A waveform library was created using the new AC-DC EPG monitor with different levels of input resistance (Ri). Six different waveforms were recorded and correlated via histological studies and grouped into three phases: non-probing waveforms (Z and Np), pathway waveforms (Tl1), and ingestion waveforms (Tl2 and Tl3). The Z waveform was observed when the stink bug was standing still on the plant surface, Np when the stink bug was walking on plant surface, Tl1 was associated with stylet insertion and deep penetration into the plant tissue, and Tl2 when the stink bug was feeding on xylem vessels. The Tl3 waveform was associated with the rupture of stalk cells and was divided into two subtypes (Tl3a and Tl3b). The Tl3a waveform probably represents cell laceration with combined enzymatic maceration of stalk tissues, while Tl3b represents a short ingestion period of macerated tissues. *Tibraca limbativentris* uses two strategies to feed on rice stalks: a salivary sheath for feeding on xylem vessels and cell rupture (laceration and maceration) for feeding on parenchyma cells. Our study provides crucial benchmark definitions of waveforms. Future studies can now compare effects of treatments on stink bug feeding, to ultimately improve management of this pest in rice.

The rice stalk stink bug, *Tibraca limbativentris* Stål, is an important rice pest in Brazil and several other South American countries (Pantoja et al. 1995, Fernandes 1998, Perez-Gelabert 2008). This species is also a potential invasive pest in North America (Brambila 2009). In Brazil, the rice stalk stink bug feeds on alternative (non-cultivated) host plants during the off-season (Pasisni et al. 2018). Adults and nymphs feed only on vegetative structures (such as the base of rice stalks) (Barrigossi et al. 2004). Feeding for 24 h during the vegetative plant stage dries the central shoot (dead hearts symptom) while feeding during the reproductive stage leads to malformed panicles (white panicle symptom) (Barrigossi et al. 2004).

The rice stalk stink bug can cause rice yield losses as high as 90%. During feeding, the insects cause significant plant tissue damage by injecting saliva and sucking tissue content (Ferreira et al. 1997). However, more research is needed to better understand their feeding behavior in rice crops. Electropenetrography (EPG) can provide an accurate way to evaluate the feeding behavior of hemipterans (Walker 2000).

In EPG, a low-voltage electrical signal flows through the sucking insect and its food source because they are connected through the same circuit. The system generates waveforms on a computer screen that represent different steps of the feeding activity: stylet penetration, salivation, ingestion, and non-probing activities such as standing still or walking on the plant surface (Tjallingii 1978, Walker 2000).

The new AC-DC EPG allows variable resistance inputs that range from 10^6^ to 10^13^ Ohms (Backus and Bennett 2009). Variable input resistance makes it possible to determine important waveform characteristics such electrical origins (resistance [R] component and electromotive force [emf] component) by interpreting changes in waveform appearance. R is the physical resistance to the applied electrical signal conveyed by ionized fluids moving through the stylets, while emf is biopotentials generated independent of the applied signal, which develops as ionic fluids pass through thin capillary tubes such as stylets (Backus et al. 2016). The R component is greater at low Ri levels while the emf component is more emphasized at high Ri levels (Backus and Bennett 2009). Associating electrical components with histological aspects will shed light on the biological meanings of the waveforms (Cervantes and Backus 2018, Lucini and Panizzi 2018b).

EPG was first used to study the feeding behavior of aphids (McLean and Kinsey 1964); however, in recent years, this technique has also been used to evaluate the feeding behavior of other piercing-sucking insects, such as leafhoppers (Joost et al. 2006), psyllids (Bonani et al. 2010), and pentatomids (Lucini and Panizzi 2016). The feeding behavior of several pentatomid species has been described using EPG: *Edessa meditabunda* (F.) in soybeans (Lucini and Panizzi 2016), *Piezodorus guildinii* (Westwood) in soybeans (Lucini et al. 2016), *Dichelops melacanthus* (Dallas) in maize (Lucini and Panizzi 2017a), *Dichelops furcatus* (F.) in wheat (Lucini and Panizzi 2017b), *Euschistus heros* (F.) in soybeans (Lucini and Panizzi 2018a), *Nezara viridula* (L.) in soybeans (Mitchell et al. 2018), and *Halyomorpha halys* Stål in broad beans (Vicia faba L.) (Serteyn et al. 2020). According to Lucini and Panizzi (2018b), EPG has great potential for increasing our understanding of the feeding process of stink bugs, which may in turn lead to new ways of mitigating impacts on crops, and potential management strategies such as selecting plants with greater insect resistance.

We investigated the feeding behavior of *T. limbativentris* in rice plants by building and characterizing an EPG waveform library. We also associated specific feeding sites with histological information and proposed biological meanings of each recorded waveform.

## Materials and Methods

### *Tibraca* limbativentris Rearing and Rice Plant Sourcing

The experiments were conducted at the Entomology Laboratory of the Goiano Federal Institute, Urutaí Campus (Urutaí, Goiás, Brazil). Adult *T. limbativentris* specimens were collected in the field, kept in cages, and fed on rice plants (cv. BR IRGA 409) for oviposition. The eggs were then collected and transferred to a plastic container (11 × 11 × 3.5 cm) lined with a moistened paper towel and kept in the laboratory (T 25 ± 2°C, RH 70 ± 10%, 14-h photoperiod) until hatching. The nymphs were maintained under the same conditions until reaching the second instar and were then transferred to rice plants (same cultivar, covered with *voile* mesh) until reaching the adult oviposition phase.

BRS A502 (commercial cultivar) seeds were sown weekly in soil (0.5-liter plastic pots) and kept in a greenhouse (T 30 ± 5°C, RH 70 ± 10%, 12-h photoperiod). After reaching the V5 stage (42 d after emergence) (Counce et al. 2000), the plants were separated and taken to the laboratory to be used once in the EPG recordings.

### Insect Wiring and EPG Recordings

Adult *T. limbativentris* females (~12 d) were separated from the colony and starved (without water) for 18 h before wiring. Next, the insects were attached to a 4-cm gold wire (0.1 mm in diameter) with a loop at one end, according to the methodology of Lucini and Panizzi (2016). A new AC-DC EPG monitor (Backus and Bennett 2009, Backus and Shih, in press; EPG Technologies, Inc., Gainesville, FL) was used to record feeding activity for 8 h uninterrupted. The experiment was performed under laboratory conditions (25 ± 2°C, RH 70 ± 10%) and continuous luminosity. The insects were individually connected to each EPG head stage amplifier and each was placed on its own rice stalk (V5 stage) (Counce et al. 2000). A copper wire (plant electrode) was inserted into the soil to create an electrical circuit within a Faraday cage.

The waveforms were recorded and digitized at a sample rate of 100 Hz per channel using a WinDaq DI-710 (Dataq Instruments, Akron, OH) connected to a computer running WinDaq Lite software (Dataq). An offset control was used to avoid rectifier fold-over and retain native waveform polarity after rectification (Backus and Shih, in press). The waveforms were categorized according to appearance and electrical characteristics (frequency, relative amplitude, and deduced electrical origin) (Almeida and Backus 2004). The waveforms were named using the convention of Lucini and Panizzi (2016) with a Tl, for *T. limbativentris*, a number for wave type and a lower-case letter for subtype. The same naming convention has been used in other studies on stink bugs (Lucini et al. 2016, Mitchell et al. 2018, Serteyn et al. 2020).

### Experimental Design

The EPG recordings were made using 50 mV AC applied signal with four different input resistance (Ri) levels: 10^6^, 10^7^, 10^8^, and 10^9^ Ohms. Sixty-two stink bugs were successfully recorded (10^6^*n* = 16, 10^7^*n* = 16, 10^8^*n* = 14, 10^9^*n* = 16). The electrical origin of each waveform and associations between biological meaning and waveforms can be determined by using different Ri levels. The R component is more emphasized at low Ri levels (10^6^ Ohms), while the emf component is more emphasized at high Ri levels (10^9^ Ohms) (Backus et al. 2019). At 10^7^ and 10^8^ Ohms, R and emf components tend to be evenly balanced (Lucini and Panizzi 2018b). As suggested by Cervantes and Backus (2018), 12 recordings (insects) at 10^7^ and 10^9^ Ohms were individually analyzed to obtain the number and duration of each waveform. The waveforms were recorded using Windaq Waveform Browser software (Dataq). To obtain the frequency and relative amplitude of the waveforms, 20 recordings (insects) at 10^7^ (*n* = 10) and 10^9^ (*n* = 10) Ohms were analyzed as suggested by Cervantes and Backus (2018). Four nonsequential EPG variables were calculated: WDI (waveform duration per insect), NWEI (number of waveform events per insect), WDEI (waveform duration per event per insect), and PRT (percentage of recording time) (Backus et al. 2007).

### Histological Investigations

Histological studies were conducted with methods from Lucini and Panizzi (2016) to correlate the stylet/salivary sheath positions on the rice stalk with the AC-DC EPG probing waveforms. Specifically, a set of *T. limbativentris* females was recorded at 10^7^ Ohms Ri and 50 mV AC, when insects were probing R1 rice plants (panicle differentiation; Counce et al. 2000). R1 plants were used because their stalks are more rigid than those of younger plants, whose overlapping leaf sheaths and gaps between leaves make it more difficult to immobilize stylets during sectioning. Furthermore, the waveforms and feeding behavior of the stink bugs were similar during both plant stages (V5 and R1).

When specific waveforms were identified on the computer screen, the EPG monitor was turned off, and the stylets were cut with micro-scissors. Next, the section of the rice stalk with the severed stylets was removed and thin tissue sections were cut using a sharp razor blade (Wilkinson Sword, London, United Kingdom). Sections were then mounted on semi-permanent slides and were viewed under a stereomicroscope (Bel Photonics, Model SZT, Monza, Italy).

Stylet tip and/or the salivary sheath positions on the rice stalk were determined using 5 Tl1, 12 Tl2, and 6 Tl3a waveform samples. Images of the histological sections were then captured using a microscope (Bel Photonics, Model Bio SSI, Monza, Italy) coupled to a Bel videocamera (Eurekan) and a computer.

### Statistical Analysis

A generalized linear model (GLM) for Poisson response and log link was fitted to the count data (NWEI) to compare waveforms through the analysis of deviance (chi-squared test) and pairwise comparisons using the *z*-test. GLMs for Gamma responses with log link were fitted for the duration variables (WDI, WDEI, and PRT), with the same purpose. The statistical analyses were performed in R version 4.0.1 (www.rproject.org). Differences were considered significant at α = 0.05.

## Results

### Overview of EPG Waveforms for *T. limbativentris*

**Fig. 1.**
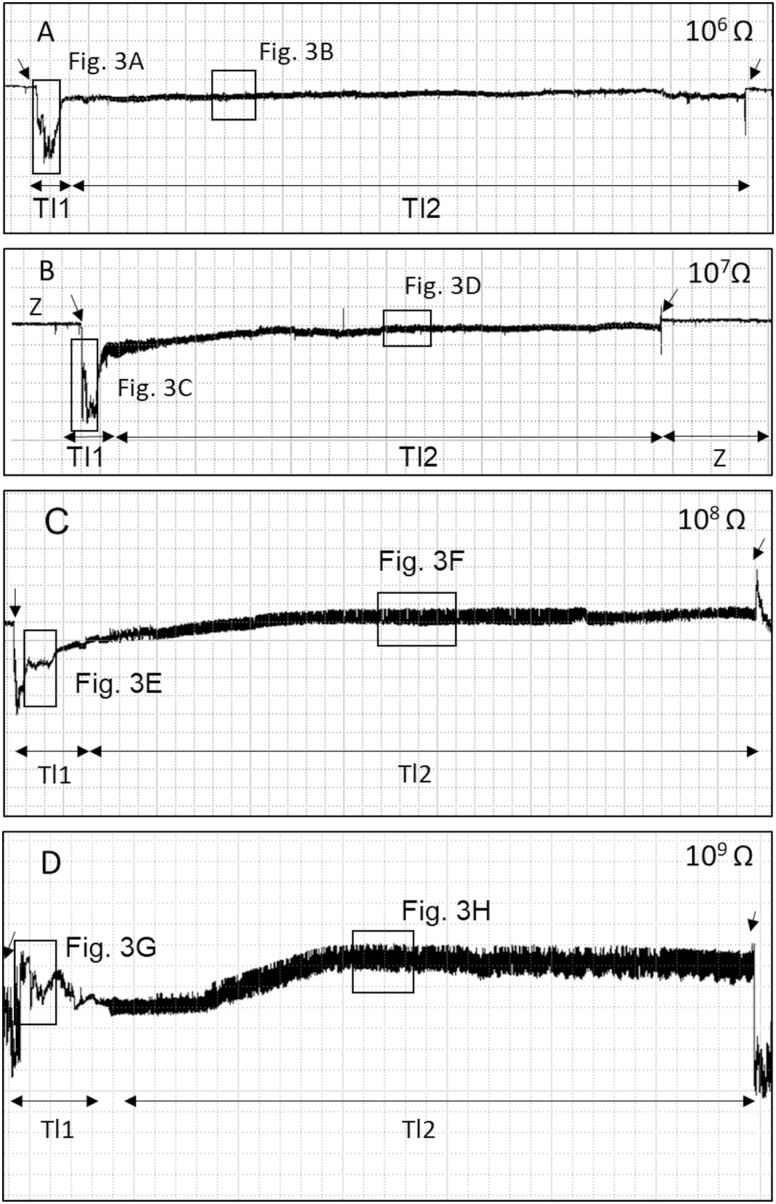
Overview of EPG waveforms (Tl1 and Tl2) from *Tibraca limbativentris* on rice stalk at 10^6^ Ohms (A), 10^7^ Ohms (B), 10^8^ Ohms (C), 10^9^ Ohms (D), and 50 mV AC applied signal. Coarse structure of waveforms observed with Windaq compression 400 (80 s/vertical div.), and gain 16× (A and B); 8× (C and D). Arrowheads indicate beginning or end of a probe.

**Fig. 2.**
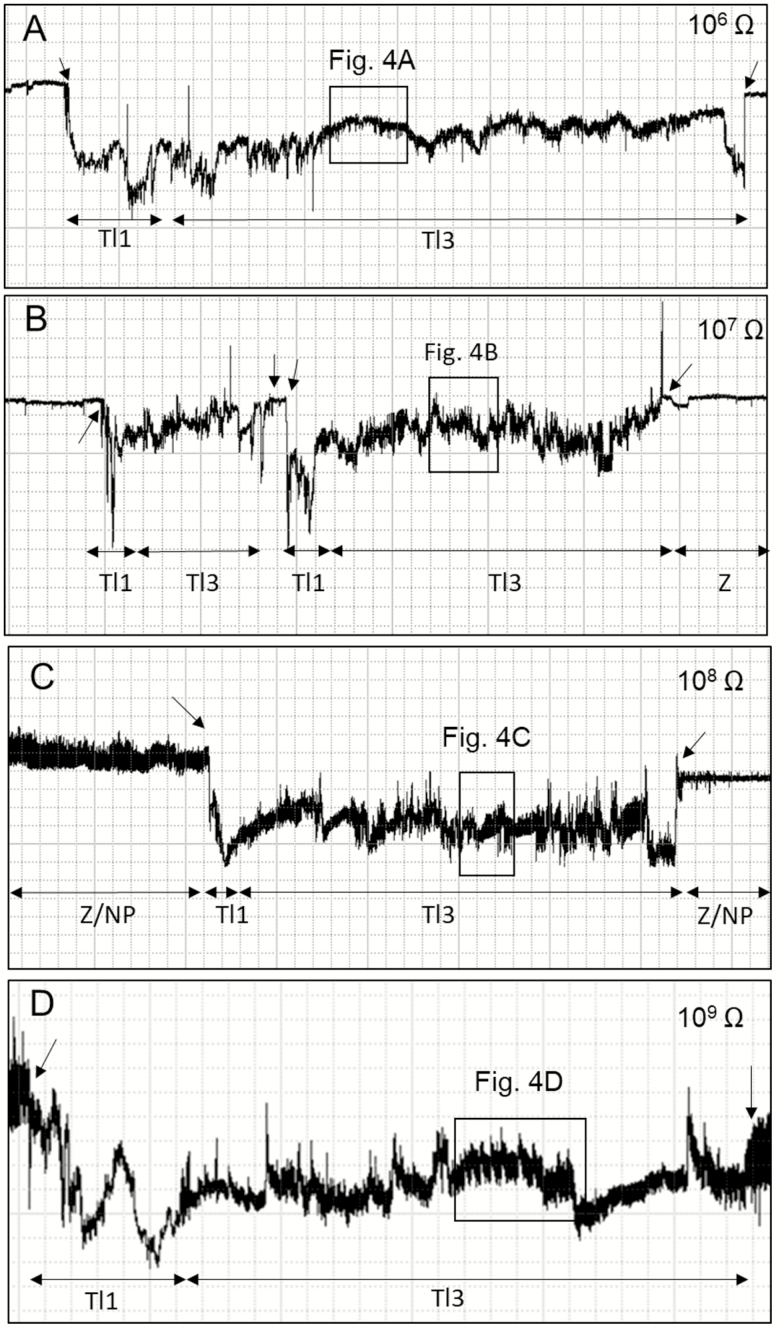
Overview of EPG waveforms (Tl1 and Tl3) from *Tibraca limbativentris* on rice stalk at 10^6^ Ohms (A), 10^7^ Ohms (B), 10^8^ Ohms (C), 10^9^ Ohms (D), and 50 mV AC applied signal. Coarse structure of waveforms observed with Windaq compression: 200 (40 s/vertical div.), gain 16× (A); 600 (120 s/vertical div.), gain 16× (B); and 400 (80 s/vertical div.), gain 16× (C and D). Arrowheads indicate beginning or end of a probe.

Both non-probing and probing waveforms were recorded for *T. limbativentris* feeding. Six different waveforms were identified and grouped in the following three phases: non-probing waveforms (Z and Np), pathway (Tl1), and ingestion (Tl2 and Tl3) (Table 1). Figures 1 and 2 summarize the waveforms recorded over the Ri range from 10^6^ to 10^9^ Ohms.

**Table 1.** Summary of EPG AC-DC waveforms, their main characteristics, and proposed biological meanings for each waveform recorded during feeding behavior of *Tibraca limbativentris* on rice stalk

Phase	Family	Type or subtype	Relative amplitude (%) (range)		Frequency (Hz) (range)		Electrical origin	Suggested biological meaning
			10^7^	10^9^	10^7^	10^9^		
Non-probing	–	Z	Flat	Flat	–	–	–	No movement on the stalk surface
		Np	Low	Medium–high	–	–	Mostly emf, some R	Walking on the stalk surface
Pathway	P	Tl1a	100	100	Irregular	Irregular	R dominated; some emf	Stylet penetration and salivary sheath secretion
Ingestion	I	Tl2	26 (10–46)	48.3 (17–96)	4.1 Hz (3.3–5.1)	3.8 Hz (3.1–5.3)	Mixed; peak = mostly R; wave = mostly emf	Xylem sap ingestion
Salivation/ ingestion	I	Tl3a	58 (20–100	47 (39–55)	Mostly irregular + burst regular sections (3.3 Hz [2.9–3.7])	Mostly irregular + burst regular sections (4.4 Hz [4.1–4.8])	R/emf	Cell laceration enzymatic maceration of stalk tissues
Ingestion	I	Tl3b	11 (8–16)	21 (19–28)	4.2 Hz (3.9–5.0)	5.0 Hz (4.4–5.7)	mostly emf	Short ingestion of macerated tissues

### Waveform Polarity

Each probe was monophasic negative at 10^6^ and 10^7^ Ohms because all waveforms occurred below the baseline. At higher Ri levels (10^8^ and 10^9^ Ohms), the waveforms became biphasic. In other words, the waveforms were initially negative but gradually rose to just above the baseline (positive) during ingestion. Thus, monophasic polarity is an R component, while biphasic polarity is an emf component. The recordings showed that none of the pathway waveforms were positive.

### Non-Probing Waveforms (Z and Np)

During non-probing, two waveforms were recorded and visually correlated with stink bug activity as the measurements were taken. The Z waveform was observed when the stink bug was not moving on the plant surface (Figs. 1B and 2B). The amplitude of the Z waveform was extremely low, did not visibly change at different Ri levels, and therefore provided a baseline during recording.

The Np waveform was similar to Z but showed irregular peaks at different Ri levels that became more pronounced at higher Ri levels (10^8^ and 10^9^ Ohms – medium to high amplitude at 10^9^ Ohms) (Table 1). The Np waveform was visually correlated with the stink bug walking on the plant surface. Non-probing activities accounted for 74.7% of total recording time and was significantly longer for all variables than any other waveform (Table 2).

**Table 2.** EPG nonsequential variables of *Tibraca limbativentris* on rice stalk

Waveform^*a*^	NWEI	WDI	WDEI	PRT (%)	Proposed activities
Z + Np	–	369.2 ±14.7 a	145.2 ±21.1 a	74.7 ±3.1 a	Rest/walking
Tl1	2.6 ± 0.5 a	11.7 ± 3.1 c	6.9 ± 2.0 c	2.4 ± 0.6 c	Pathway activities
Tl2	2.0 ± 0.3 a	67.6 ± 11.4 b	44.8 ± 7.6 b	14.3 ± 2.3 b	Xylem sap ingestion
Tl3a	4.6 ± 1.3 b	37.4 ± 9.5 b	5.4 ±1.0 c	7.8 ± 2.8b	Laceration/maceration of parenchyma
Tl3b	6.2 ± 2.3 b	8.1 ± 3.5 c	0.5 ± 0.3 d	2.0 ± 0.5c	Ingestion
*p* value	0.001	<0.001	<0.001	<0.001	

NWEI = number (±SE) of waveform events per insect; PRT = percentage of recording time (±SE); WDEI = waveform duration per event per insect (min ± SE); WDI = waveform duration per insect (min ± SE).

^*a*^Means followed by the same letter in each column do not differ statistically at 5% probability, according to the *z*-test.

### Probing Waveforms: Pathway Phase – P Family (Tl1)

**Fig. 3.**
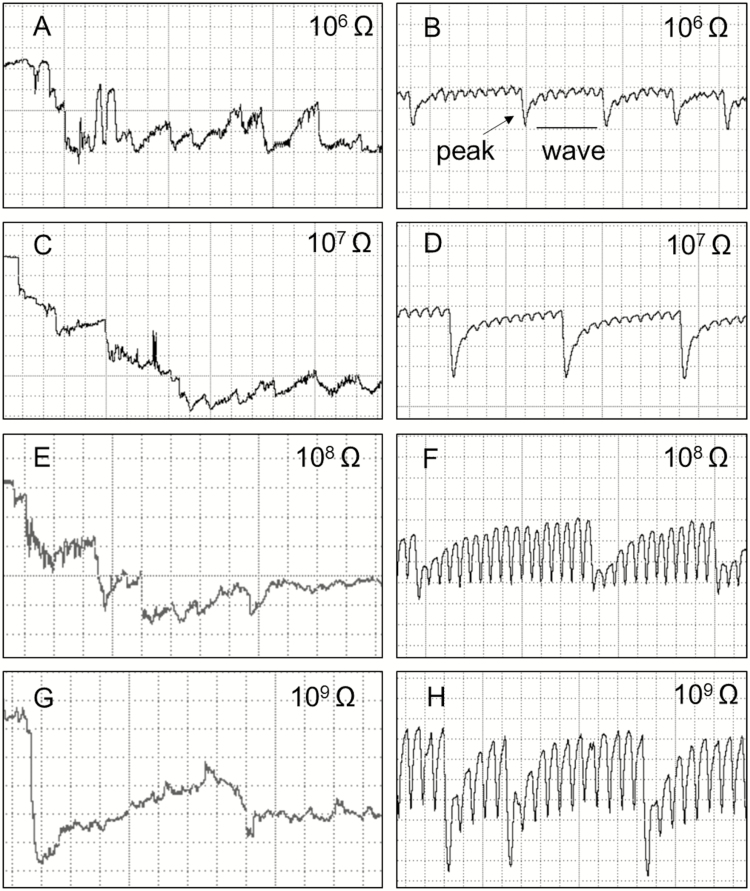
Waveforms recorded using EPG from *Tibraca limbativentris* on rice stalk at 10^6^ Ohms (A and B), 10^7^ Ohms (C and D), 10^8^ Ohms (E and F), 10^9^ Ohms (G and H), and 50 mV AC applied signal. Expanded views of waveform Tl1 (A, C, E, G) and Tl2 (B, D, F, H). Definition of peaks and wave portions (B). Coarse structure of waveforms observed with Windaq compression: 10 (2 s/vertical div.), gain 16× (A, C, E, and G); 2 (0.4 s/vertical div.), gain 32× (B, D, F, and H).

This phase consisted of only one waveform (Tl1). Tl1 was visible across all Ri levels (10^6^–10^9^ Ohms), with voltage levels decreasing abruptly from non-probing waveforms (Z and Np) (Fig. 1A–D, indicated by the arrows on the left). Tl1 had an R-dominated origin because it was more visible at low Ri levels than at high Ri levels. Nevertheless, the emf component was still present in this waveform. Tl1 also was irregular (Fig. 3A, C, E, and G) and had the highest (negative) amplitude (100%) of any of the recorded waveforms (Table 1).

Tl1 it always appeared before Tl2 (stylets inserted in xylem vessels; see below) (Fig. 1) or Tl3 (Fig. 2). Tl1 was always easy to distinguish from Tl2, but sometimes difficult to distinguish from Tl3. Tl1 was a relatively brief waveform that was repeated almost three times for each insect. It also represented among the smallest proportions of total recording time and its duration (see below) (Fig. 1; Table 2).

### Probing Waveforms: Ingestion Phase – I Family (Tl2 and Tl3)

**Fig. 4.**
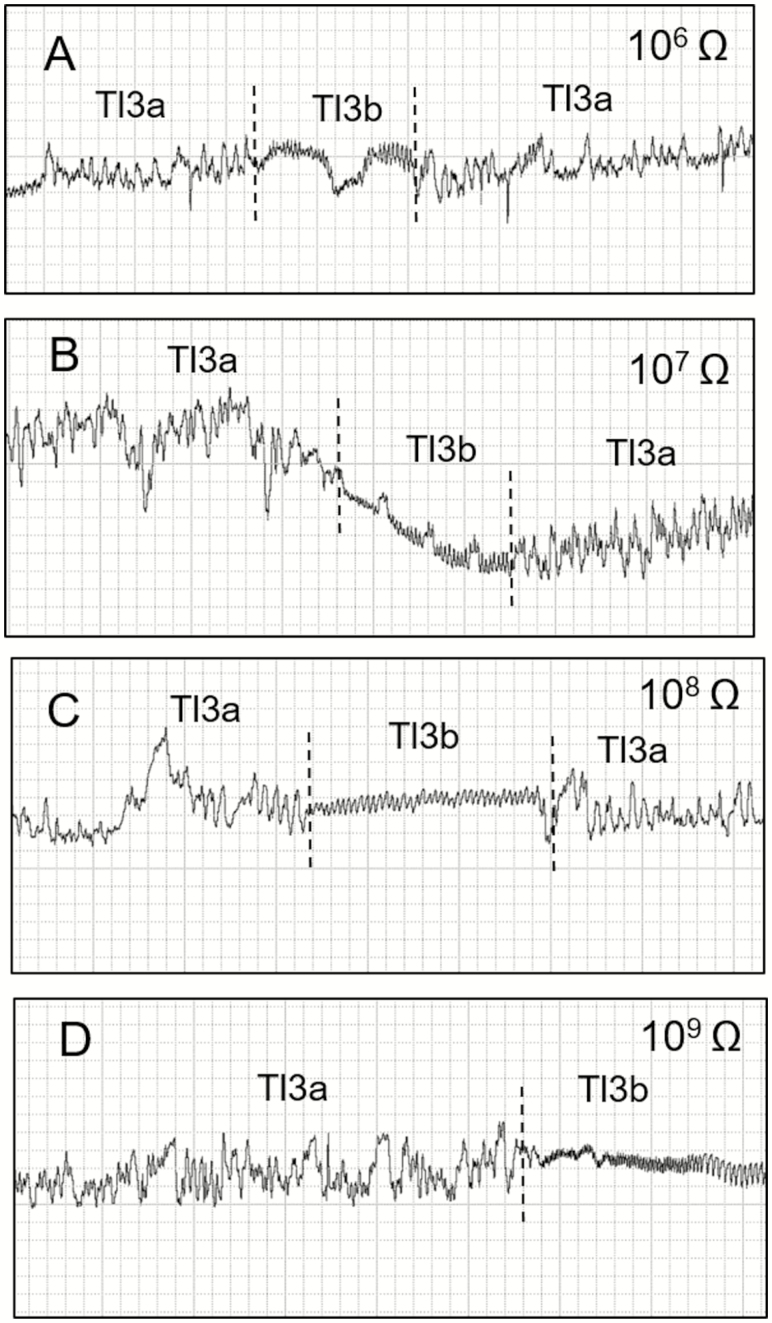
Waveforms recorded using EPG from *Tibraca limbativentris* on rice stalk at 10^6^ Ohms (A), 10^7^ Ohms (B), 10^8^ Ohms (C), 10^9^ Ohms (D), and 50 mV AC applied signal. Expanded view of waveforms Tl3a and Tl3b occurring interspersed with each other. Coarse structure of waveforms observed with Windaq compression: 4 (0.8 s/vertical div) and gain: 32× (A, B); 16× (C and D).

This family of waveforms consisted of Tl2 and Tl3 and two Tl3 subtypes (Tl3a and Tl3b) (Table 1; Figs. 3 and 4).

#### Type Tl2

Tl2 was always preceded by Tl1 (Fig. 1). The waveform was regular, and with low frequencies that were very similar among Ri levels (Table 1). Tl2 was interspersed with regularly distributed and mostly negative-oriented peaks (Fig. 3B, D, F, and H; peaks and wave portions are defined in Fig. 3B). However, peak inversions (positive-oriented) were also observed in some xylem recordings.

The electrical origin of Tl2 was a mixture of R and emf components. At low Ri (10^6^ and 10^7^ Ohms), the T12 peaks (Fig. 3B and D, respectively) had much greater amplitude than at high Ri levels (10^8^ and 10^9^ Ohms). However, the wave portion was more evident at high Ri levels than at low Ri levels (Fig. 3B, D, F, and H). Furthermore, wave amplitude increased with increasing Ri (10^7^ = 26%, 10^9^ = 48.3%) (Table 1), suggesting that the wave was dominated by the emf component, and that the peaks were R dominated.

Tl2 was visually correlated with the stylets immobile in the plant tissue. Tl2 events were repeated twice per insect (NWEI). Per-insect and per-event durations (WDI and WDEI, respectively) were the longest numerically, but were not significantly different from Tl3a (see below). Tl2 accounted for the highest percentage of total recording time (Table 2).

#### Type Tl3

Tl3 was always preceded by the Tl1 waveform (Fig. 2A–D) and was divided into two subtypes: Tl3a and Tl3b, whose occurrence alternated frequently (Fig. 4). Tl3a occurred irregularly with both positive and negative peaks within the same recording. Some Tl3a sections showed regularity (3.3–4.4 Hz at 10^7^ and 10^9^ Ohms, respectively), but only occasionally and for short periods, with a mix of R and emf components. Amplitude decreased with increasing Ri (10^7^ = 58%, 10^9^ = 47%). Tl3a was visually correlated with continuous stylet movements (in and out) within the rice stalk,

Tl3b was characterized by its short duration, regular frequency, and low amplitude (Table 1). Tl3b was more apparent at higher Ri levels than at lower ones and was therefore mostly emf-dominated. T13b was repeated more often than Tl3a (NWEI, Table 2); however, each event of Tl3b was much shorter than one of Tl3a (WDEI, Table 2). The sum of all Tl3a was much longer than Tl3b (Table 2). Tl3b occurrence was interspersed with Tl3a within short windows (30 s per event [WDEI]) and was visually correlated with moments when the stylets were immobile within the plant tissue. Tl3b had the smallest percentage of probing time, but was not significantly different from the also-short Tl1 waveform (Table 2).

### Correlation of Waveforms With Specific Feeding Sites Using Histological Studies

**Fig. 5.**
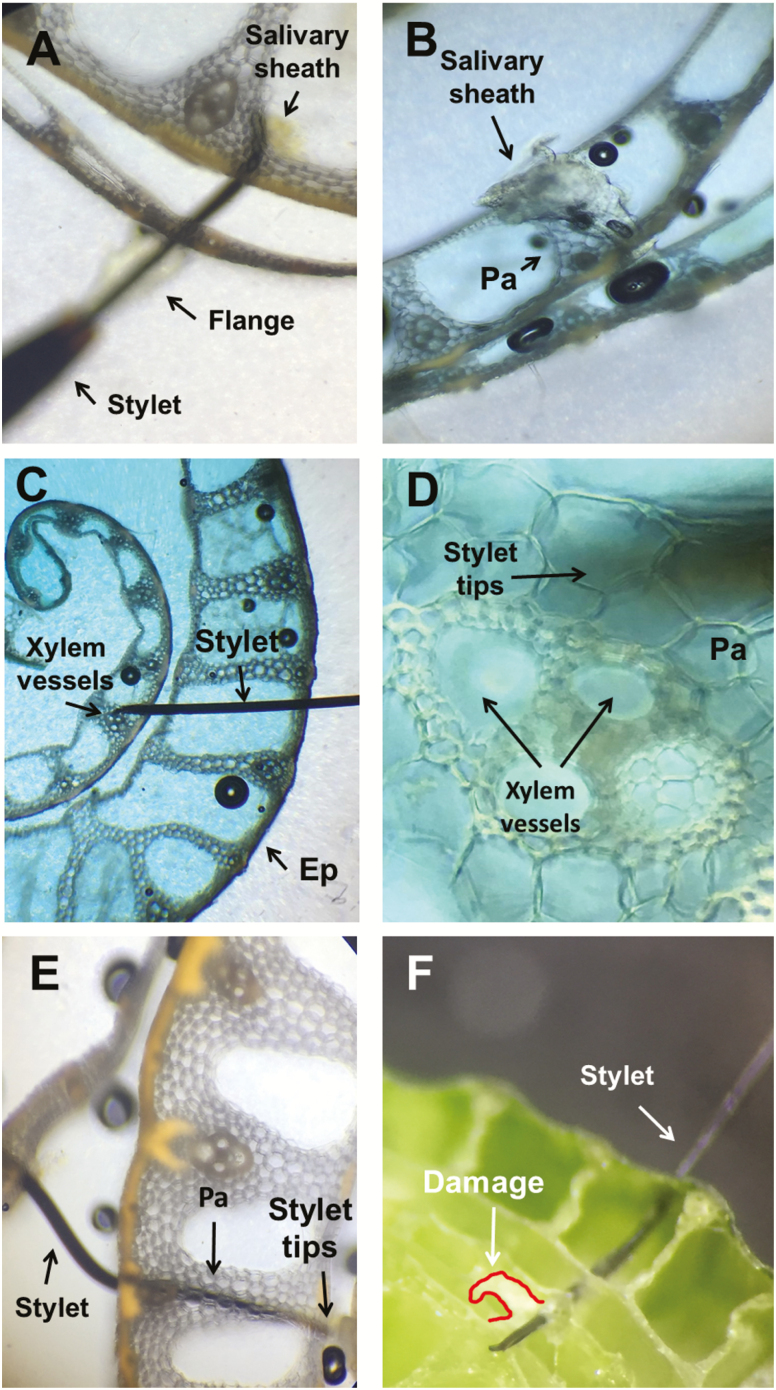
Cross-sections of rice stalk (R1 stage) containing severed stylets and salivary sheath of *Tibraca limbativentris*. Salivary sheath and stylet tips positioned in parenchyma cells during waveform Tl1 (10×) (A). Salivary sheath in parenchyma tissue during waveform Tl1 (10×) (B). Stylet tips in xylem vessel during the waveform Tl2 (4×) (C). Detail of stylet tips near the xylem vessel during waveform Tl2 (40×) (D). Stylet tip in parenchyma cells during waveform Tl3a (10×) (E). Cross-section of fresh rice stalk with stylet showing a visual damage area (white region surrounded by red line) after recording of an Tl3 event (4×) (F). Ep = epiderm, Pa = Parenchyma.

*Tibraca limbativentris* feeding activity (recorded with EPG) was correlated with histological examinations of plant tissue containing severed stylets and/or a salivary sheath (Fig. 5). During the Tl1 waveform (pathway phase), the severed stylets and salivary sheaths (*n* = 5) were positioned in parenchyma tissue (Fig. 5A and B). All histological images (*n* =12) for Tl2 showed the presence of a salivary sheath and stylet tips positioned in xylem vessels (Fig. 5C and D).

Stylet tips were found in parenchyma tissue (*n* = 7) during the Tl3 waveform (Fig. 5E). After the Tl3 waveform, stalk cross-sections showed damage (white delimited by a red line, Fig. 5F). Finally, a complete salivary sheath was formed before waveform Tl2, while only an incomplete salivary sheath was found before Tl3.

## Discussion

In the current study, the stylet penetration behavior of *T. limbativentris* in rice plants was first defined by AC-DC EPG and then a waveform library was created to characterize the blend of R and emf components in each waveform. Six EPG waveforms were identified that characterized *T. limbativentris* feeding. Other studies have used EPG to monitor stink bug feeding on plants (Lucini and Panizzi 2018b).

In general, heteropterans use two feeding strategies. One is the salivary sheath strategy, where the insect creates a complete sheath that is formed by gelling saliva around stylets while the plant tissue is penetrated. The other feeding strategy is cell rupture (lacerating and/or macerating) (Backus et al. 2005, Lucini and Panizzi 2018b). Here, the insect inserts the stylets into the plant tissue and then moves them in and out (lacerating) while secreting watery saliva to destroy cells (macerating) for subsequent ingestion (Miles 1972, Hori 2000, Backus et al. 2005). While lacerating and macerating are distinct tactics that usually occur separately, pentatomids may perform them simultaneously (Lucini and Panizzi 2018b).

Depending on the species, stink bugs may use one or both feeding strategies. *Edessa meditabunda*, for example, uses only one strategy (salivary sheath) when feeding on soybean stems (vascular tissue), while other species such as *D. melacanthus* feeding on maize seedlings (in vascular and parenchyma tissue) (Lucini and Panizzi 2017a) and *D. furcatus* feeding on wheat ear heads (Lucini and Panizzi 2017b) used both the cell rupture and sheath feeding strategies. Similarly, *Eu. herus* (Lucini and Panizzi 2018a) and *N. viridula* (Mitchell et al. 2018) feeding on soybean pods use both strategies (salivary sheath and cell rupture). We found that *T. limbativentris* also used both strategies (salivary sheath and cell rupture) while feeding on rice stalks (vegetative structure). In general, the waveforms of *T. limbativentris* were similar in appearance and biological significance to those of *D. melacanthus* feeding on maize seedlings. However, the feeding behavior of *D. melacanthus* is qualitatively different because it shifts to grains/seeds when available (Panizzi and Silva 2012),

The Z waveform represented the insect standing still on the plant surface while the Np waveform was associated with the insect walking on the plant surface. Both waveforms were recorded at all Ri levels and were visually correlated with activity type. The pathway phase was associated with only one waveform (Tl1). During this phase, the insect inserted its stylet into the plant tissue while a salivary sheath was formed that was either complete or partial, depending on feeding strategy and site. A complete salivary sheath is formed when the insect uses the salivary sheath strategy (ingesting from vascular vessels) (Bonani et al. 2010, Lucini and Panizzi 2016). An incomplete salivary sheath is formed when the insect uses cell rupture as a feeding strategy. Here, a salivary flange is formed on the surface of the plant but an attached sheath is not formed inside the plant. (Lucini et al. 2016).

Tl2 was associated with ingestion from vascular tissue, specifically xylem vessels. During T12, all histological analyses showed that the stylet tips were in xylem vessels while visual analyses showed that the stylets remained immobile within the plant tissue. The appearance and electrical origins of this waveform were similar to those of other xylem ingestion waveforms in heteropterans (Lucini and Panizzi 2017a, 2018a). According to Mitchell et al. (2018), Lucini and Panizzi (2018a) and Rivera and Mitchell (2020), insects ingest from xylem for hydration, which may explain the long ingestion periods in xylem vessels.

*Tibraca limbativentris* faced downward while feeding on the vertical rice stalk. The same behavior was observed in *D. melacanthus* while feeding on the stems of maize seedlings (Panizzi and Lucini 2019). Both insects share the same feeding strategies (cell rupture and salivary sheath), which, according to the authors, may be more effective when facing downward and against the flow of xylem sap, which travels up from the roots to the leaves.

Cell rupture was associated with the Tl3 waveform. This waveform was divided into two subtypes (Tl3a and Tl3b). Tl3a was visually correlated with continuous stylet movements (back and forth) within the rice stalk, which mechanically (laceration) and chemically (maceration through enzymes) dissolves the tissue and cell content of the rice stalk. Other pentatomids have shown similar behaviors while feeding on vegetative and reproductive structures (Lucini and Panizzi 2018b). Tl3b frequently alternated with Tl3a and was visually correlated with periods when the stylet remained immobile within the plant tissue. Other studies have shown similar stink bug behavior while feeding on vegetative (Lucini and Panizzi 2017a) and reproductive structures (Lucini et al. 2016; Lucini and Panizzi 2017b, 2018a). Thus, Tl3a represented the laceration/maceration preparation behaviors, while Tl3b (regular waveform and frequency) were short and represented the ingestion of cell content that was degraded during Tl3a. The destruction of the stalk tissue may be related to ‘dead-heart’ and ‘white panicle’ symptoms that result from long feeding periods. During the vegetative plant stage, stink bugs remain on the plant for 24 h, which is sufficient to kill the center shoot and create watery lesions on the stalk (Barrigossi et al. 2004).

Most pentatomids feed on immature seeds (Panizzi and Silva 2012). Exceptions include *T. limbativentris* and *E. meditabunda* that feed exclusively on vegetative structures. *Edessa meditabunda* ingests from both xylem vessels and phloem sieve elements as it feeds on soybean plants (Lucini and Panizzi 2016). Our experiments with adult *T. limbativentris* did not show waveforms associated with ingesting phloem sap. Instead, these insects probably obtained essential nutrients by macerating/lacerating the parenchyma tissue. We found that *T. limbativentris* used two strategies to feed on rice stalks: a salivary sheath when ingesting from xylem vessels and cell rupture via laceration and maceration when salivating and ingesting the content of parenchyma cells. These findings may contribute to the development of management strategies via quantitative studies that compare the effects of different treatments on feeding, the selection of insect resistant plants, and the effects of different insecticides.
